# Genetic diversity of human respiratory syncytial virus isolated among children with acute respiratory infections in Southern Cameroon during three consecutive epidemic seasons, 2011–2013

**DOI:** 10.1186/s41182-018-0088-7

**Published:** 2018-04-03

**Authors:** Sebastien Kenmoe, Marie-Astrid Vernet, Fabien Miszczak, Julia Dina, Matthieu Schoenhals, Véronique Penlap Beng, Astrid Vabret, Richard Njouom

**Affiliations:** 1 Virology Department, Centre Pasteur of Cameroon, P.O.Box 1274, Yaounde, Cameroon; 2 Normandie Université, 14032 Caen, France; 3 UNICAEN, UNIROUEN, GRAM, 14000 Caen, France; 4 Department of Virology, University Hospital of Caen, 14000 Caen, France; 5 Département de Biochimie, Université de Yaoundé I, BP 812 Yaoundé, Cameroon

**Keywords:** Cameroon, Genetic characterization, Human respiratory syncytial virus, Molecular epidemiology

## Abstract

**Background:**

Human respiratory syncytial virus (HRSV) is the main viral cause of severe lower respiratory tract disease in infants and young children. The aim of this study was to describe for the first time the genetic variability of HRSV in Cameroonian patients living in Yaounde for three consecutive epidemic seasons.

**Methods:**

HRSV-positive nasopharyngeal samples detected in children less than 15 years in Yaounde were collected from September 2011 to December 2013. Semi-nested RT-PCR, sequencing, and phylogenetic analyses of the second hypervariable region of the *G* gene were performed.

**Results:**

A total of 57 HRSV-positive samples were collected during the study period. Among these, 46 (80.7%) could be amplified in the *G* gene. HRSV group A (HRSV-A) and group B (HRSV-B) co-circulated in this population at 17.4 and 82.6%, respectively. HRSV-A strains clustered in the NA-1 genotype while HRSV-B strains clustered in the BA-9 genotype. HRSV-A strains accounted for 33.3% (2/6), 4.3% (1/23), and 29.4% (5/17) of the viruses isolated in 2011, 2012, and 2013, respectively.

**Conclusions:**

This study reports molecular epidemiology data of HRSV in Cameroon for the first time. Additional studies are required to clarify evolutionary patterns of HRSV throughout sub-Saharan Africa to support antiviral and vaccine development.

## Background

Human respiratory syncytial virus (HRSV) is the major viral cause of bronchiolitis and pneumonia in infants and young children worldwide. On the basis of a meta-analysis, Nair and his colleagues found an incidence of 33.8 million (95% CI, 19.3 to 46.2) cases of acute lower respiratory infections associated with HRSV each year in children under 5 years [1]. About 66–199 thousand deaths were recorded in this meta-analysis. A total of 91% of hospitalizations and nearly all deaths (99%) were registered in developing countries. According to the recent classification of the International Committee on Taxonomy of Viruses, HRSV belongs to the family *Pneumoviridae* and a genus *Orthopneumovirus* [2]. This virus is an enveloped negative-sense single-stranded RNA virus. Its genome is non-segmented and counts about 15,200 nucleotides. The envelope contains two main surface glycoproteins: the attachment protein (G) and the fusion protein (F), which are responsible for the attachment and viral entry into the host cell, respectively. The F protein is relatively conserved while the main differences between HRSV-A and HRSV-B are located on the G proteins. The latter has a length varying from 282 to 321 amino acids (AA) according to the genotypes and contain a cytoplasmic domain, a transmembrane domain, and an ectodomain. Cytoplasmic and transmembrane domains are conserved while the ectodomain has two hypervariable regions (HVR1 and HVR2), separated by a highly conserved central segment comprising the 13 amino acids (AA 164–176) receptor binding site [3, 4]. Due to its high variability, the HVR2 domain represents the main target for evolutionary and molecular epidemiology studies of HRSV [5–15]. Two major groups of HRSV (HRSV-A and HRSV-B) have been distinguished based on molecular and serological techniques [16]. Each subgroup is further categorized into genotypes based on the nucleotide sequence variation within the third hypervariable region of heavily glycosylated G-glycoproteins. There are 14 genotypes for RSV-A (GA1–7, SAA1, NA1–4, CB-A, and ON1) and 29 genotypes for RSV-B (GB1–5, SAB1–4, URU1–2, CB-1, CB-B, BA-C, THB, and BA1–14) [5, 6, 9, 17–29]. Despite huge efforts, no approved vaccine is available for the prevention of HRSV infection [30]. Nevertheless, more than 60 vaccine development programs are under various stages, and some of them could be used in the near future. Additional studies on HRSV genetic variability will support the development of new vaccines. To date, characterization of HRSV strains into groups and genotypes has not been performed in many African sub-Saharan countries [31, 32]. An initial study in Cameroon showed that HRSV circulated in the rainy season from October to December at 5.7% in outpatients with influenza-like illness visiting influenza surveillance centers in 2009 [33]. We have recently shown that HRSV was the second most common respiratory virus (13.3%) after human adenovirus in children hospitalized in Yaounde, Cameroon [34]. We examine here the genetic variability of the second hypervariable region of *G* gene (HVR2) of HRSV strains detected during three consecutive epidemic seasons (2011–2014) in Cameroonian children.

## Methods

### HRSV samples

In this study, 822 nasopharyngeal swabs were screened for HRSV. The samples included 436 inpatient and 386 outpatients recruited from September 2011 to October 2014 in the pediatrics unit of the “Centre Hospitalier d’Essos” in Yaounde, Cameroon. Fifty-seven HRSV-positive nasopharyngeal swabs collected from September 2011 to December 2013 were included in the current study. The median age of study participants was 13 months IQR [6–21.5]. The age of patients ranged from 1 month to 4 years. The female/male ratio was 1.1 (30/27). Samples included 47 (82.5%) inpatients and 10 (17.5%) outpatients. Samples were submitted to the “Centre Pasteur du Cameroon” as part of influenza surveillance in Cameroon and the IMMI (Institut de Microbiologie et de Maladies Infectieuses) project. Written informed consent was obtained from the parents or guardians of all children enrolled in the study. The procedures of the original study were evaluated and approved by the National Ethics Committee and the Ministry of Public Health of Cameroon.

### RNA extraction, HRSV *G* gene amplification, and sequencing

RNA was extracted from HRSV-positive nasopharyngeal swabs as previously described [34], and the HRSV *G* gene was amplified by using semi-nested RT-PCR with primers targeting the HVR2 region of the *G* gene [10, 35]. The RT-PCR reaction was performed with the SuperScript® III One-Step RT-PCR System (Thermo Fisher Scientific, Carlsbad, CA, USA) following the manufacturer’s instructions. Briefly, 5 μL of extract was added to 45 μL of PCR reaction mixture containing ABG490 forward and F164 reverse primers. The primer ABG490 corresponds to the positions 497–519 of the *G* gene sequences of the A2 reference sequence (M11486). The primer F164 is located at positions 164–186 nucleotides of the F gene sequences on reference strain A2. A semi-nested amplification reaction was then performed with Taq DNA Polymerase (Thermo Fisher Scientific, Carlsbad, CA, USA) according to the manufacturer’s instructions. Briefly, 2.5 μL of the DNA of the RT-PCR was added to 45 μL of PCR reaction mixture containing forward primers [AG655 for HRSV-A and BG517 for HRSV-B] and F164 reverse primer. The primer AG655 is at positions 655–674 of the *G* gene of the A2 reference strain and BG517 at the positions 517–538 of the reference strain CH18537 (M17213). Fragments amplified in the semi-nested PCR were bi-directionally sequenced by Sanger method using BigDye Terminator v3.1 Cycle Sequencing kit (Thermo Fisher Scientific, Foster City, CA, USA). Sequencing was done on Applied Biosystems 3500 Series Genetic Analyzers. The primers used for sequencing were the same as those used for the semi-nested PCR.

### Sequence alignment and phylogenetic tree construction

The phylogenetic analysis was performed on the HVR2 region of the HRSV genome. Sequences from this study and those from other countries have been aligned using the Clustal W algorithm in MEGA software version 6 [36, 37]. The best substitution model was determined using MEGA-6. A phylogenetic tree was constructed with the maximum likelihood method under the Hasegawa-Kishino-Yano model with the site heterogeneity gamma model for HRSV-A and Tamura-Nei model with the site heterogeneity gamma model for HRSV-B. The robustness of the tree was evaluated using 1000 bootstrap resampling. The phylogenetic analysis was performed using MEGA software version 6. Potential *N*-glycosylated sites (NXT, where X is different from proline) and *O*-glycosylated sites have been predicted using NetNGlyc 1.0 and NetOGlyc 4.0 Server, respectively. The *O*-glycosylated sites were predicted using a score > 0.5 [38]. Positively and negatively selected sites on the HRV2 fragment were identified using single-likelihood ancestor counting (SLAC), random effects likelihood (REL), and fixed effects likelihood (FEL) methods [39]. Selective pressure analysis was performed on the Datamonkey website interface. The level of significance was chosen at 1%. The nucleotide sequences of this study were submitted to GenBank and registered with the accession numbers KU928193 to KU928238.

## Results

### HRSV genotyping

**Fig. 1:**
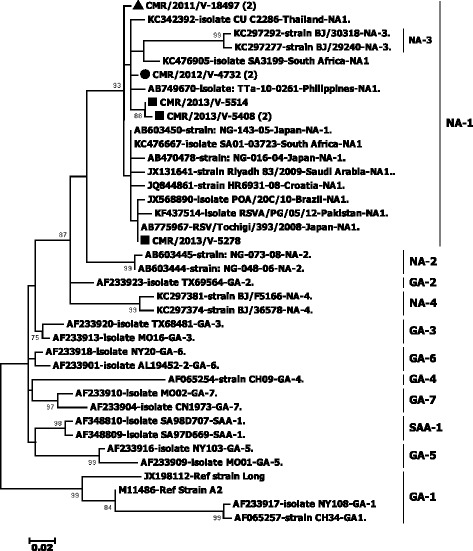
Phylogenetic trees of HRSV-A strains detected in Cameroon from 2011 to 2013. Multiple sequence alignment was performed with Clustal W. The unrooted trees were generated based on nucleotide sequences of the C-terminal HVR2 region of the *G* gene using the maximum likelihood method under the Hasegawa-Kishino-Yano model with the site heterogeneity gamma model in MEGA version 6. The scale bars represent the frequency of nucleotide substitutions, and the numbers at the nodes of the branches are the values determined for the bootstrap resampling after 1000 iterations. Only bootstrap values > 70% are presented. The reference sequences from different continents, obtained from GenBank, are identified from left to right with accession numbers, names of the strain, countries, and genotypes. Current study strains are identified from left to right by CMR (Cameroon), the year of detection, and the laboratory number. The sequences of Cameroon are marked with triangle, circle, and square for the year 2011, 2012, and 2013, respectively. The groups/genotypes assigned to branches are at the right after the bars

**Fig. 2:**
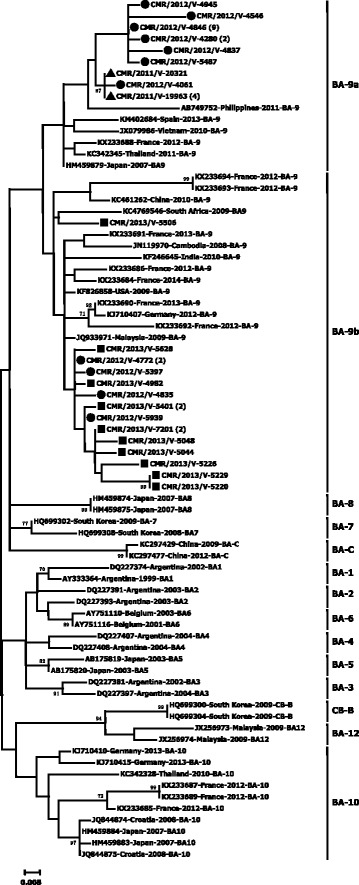
Phylogenetic trees of HRSV-B strains detected in Cameroon from 2011 to 2013. Multiple sequence alignment was performed with Clustal W. The unrooted trees were generated based on nucleotide sequences of the C-terminal HVR2 region of the *G* gene using the maximum likelihood method under the Tamura-Nei model with the site heterogeneity gamma model in MEGA version 6. The scale bars represent the frequency of nucleotide substitutions, and the numbers at the nodes of the branches are the values determined for the bootstrap resampling after 1000 iterations. Only bootstrap values > 70% are represented. The reference sequences from different continents, obtained from GenBank, are identified from left to right with accession numbers, countries, years of virus collection, and genotypes. Current study strains are identified from left to right by CMR (Cameroon), the year of detection, and the laboratory number. The sequences of Cameroon are marked with triangle, circle, and square for the year 2011, 2012, and 2013, respectively. The groups/genotypes assigned to branches are at the right after the bars

A total of 57 HRSV-positive samples were included in this study. HRSV-positive samples from this study were detected during the rainy season from September to December. Forty-six (80.7%) samples could be amplified and sequenced in the *G* gene including 6 in 2011, 23 in 2012, and 17 in 2013. The mean cycle thresholds of amplified samples were significantly lower than unamplified ones (23.9 ± 5.1 vs 31.9 ± 5.4; *p* < 0.001). HRSV-B (82.6%) predominated on HRSV-A (17.4%) in this study (Table 1). Distribution of HRSV groups varied according to the epidemic season: In 2011, HRSV-A represented 33.3% (2/6) and HRSV-B 66.7% (4/6). In 2012 and 2013, HRSV-B predominated on HRSV-A strains. HRSV-B accounted for 95.7% (22/23) and 70.6% (12/17) of strains detected in 2012 and 2013, respectively. HRSV-A strains clustered in the NA-1 genotype while HRSV-B strains clustered in the BA-9 genotype (Figs. 1 and 2). BA-9 genotype was further classified into two sub-branches that were labeled as subtypes BA-9a and BA-9b. BA-9a and BA-9b were detected in the 2011/2012 and 2012/2013 epidemic seasons, respectively. Of the 46 samples successfully amplified and sequenced, 82.6% (38/46) were hospitalized patients while 17.4% (8/46) were ambulatory patients. The majority (89.5%, 34/38) of hospitalized patients were infected with HRSV genotype BA9 (Table 2).

**Table 1:** Distribution of respiratory syncytial virus genotypes in Yaounde, Cameroon, 2011–2013

	2011	2012	2013	Total
HRSV-A (NA1)	2 (33.3)	1 (4.3)	5 (29.4)	8 (17.4)
HRSV-B (BA9)	4 (66.7)	22 (95.7)	12 (70.6)	38 (82.6)

**Table 2:** Distribution of respiratory syncytial virus genotypes according to hospitalization status in Yaounde, Cameroon, 2011–2013

	Total	Inpatients	Outpatients	*p* value
NA-1	8	4 (50)	4 (50)	< 0.01
BA-9	38	34 (89.5)	4 (10.5)	

Data are numbers, and the percentages are in parentheses

### Analysis of deduced amino acid sequences

**Fig. 3:**
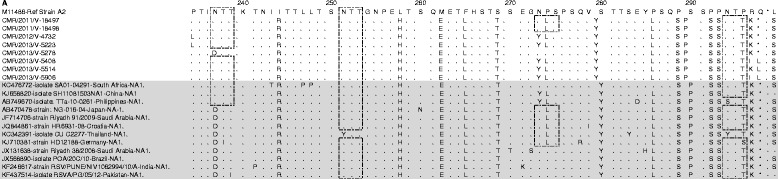
Alignment of deduced amino acid of the second hypervariable region of the *G* gene from the sequences of HRSV-A. The alignments are presented in reference to strain A2 (M11486, positions 234–300). Identical residues are represented by dots and stop codons by asterisks. The lack of amino acid is represented by dashes. The light gray shading delineates the borders of Cameroonian sequences. Potential *N*-glycosylation sites (NXT/S, where X is not proline) are shown by light dashed rectangles. Reference sequences are identified from left to right by GenBank accession number, the name of the strain, country, and genotype. Cameroonian sequences are named from left to right by CMR (Cameroon), year of detection, and lab number

**Fig. 4:**
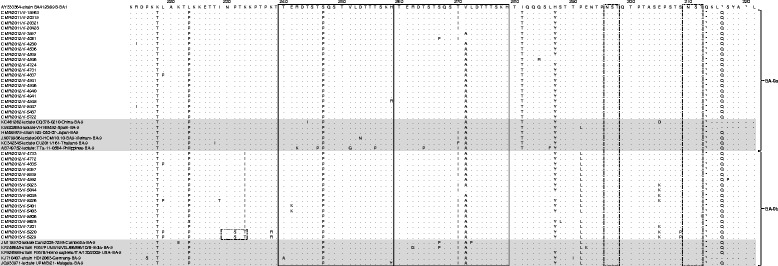
Alignment of deduced amino acid of the second hypervariable region of the *G* gene from the sequences of HRSV-B. The alignments are presented in reference to BA4128/99B prototype strain (AY333364, positions 213–321). Potential *N*-glycosylation sites (NXT/S, where X is not proline) are shown by light dashed rectangles. Identical residues are represented by dots and stop codons by asterisks. The lack of amino acid is represented by dashes. The two copies of the duplicated region of 20 amino acids specific for BA genotype are represented by simple rectangles. Genotypes/subgroups are shown on the right. The light gray shading delineates the borders of Cameroonian sequences. Reference sequences are identified from left to right by their GenBank accession number, the name of the strain, country, and genotype. Cameroonian sequences are named from left to right by CMR (Cameroon), year of detection, and lab number

The HVR2 of 8 NA-1 strains of this study was aligned with the NA-1 genotype from other countries and with the reference strain A2 (M11486) (Fig. 3). Unfortunately, we were able to retrieve only a few sequences from African countries, as data are scarce. Five NA-1 nucleotide sequences of this study had a premature stop codon (Q298Stop) that resulted in a sequence of 64 amino acids. The substitutions N237D, S269T, P274L, P286L, P289S, P292S, R297K, and Q298stop, previously reported as specific to NA-1 genotype [5, 12, 21, 35, 40], were also found in the NA-1 sequences of the current study. The HVR2 of 38 BA-9 strains of the present study were aligned with sequences from other countries and the BA4128/99B reference sequence (Fig. 4). Thirty-six BA-9 nucleotide sequences of this study had a nucleotide mutation that led to the occurrence of a premature stop codon (Q313Stop), resulting in a sequence of 100 amino acids. Two remaining Cameroonian sequences [CMR/2012/V-4772 and CMR/2013/V-4982] have a length of 106 amino acids due to a mutation that has led to the replacement of the BA4128/99B stop codon by glutamine at position 316. As previously described, substitutions K218T and S247P were specific to all BA-9 genotypes [28]. BA-9 genotype-specific mutations, T270I, V271A, and H287Y, reported by previous studies were also recorded during this work [6, 12, 14, 23, 35]. I281T and P291L mutations previously attributed to BA-9 subgroup genotype were specific to BA-9a and BA-9b subgroup sequences, respectively [13]. The variation K233I, Q284R and P291L reported as specific to BA-13 genotype, was noted only in the Cameroonian sequences of the BA-9b genotype of this study [25]. Two sequences of the BA-9b genotype had four specific mutations: P231S, K233T, P238R, and S309P. It should be noted that the P231S change had previously been identified in BA-10 HRSV genotype [5, 40].

### Analysis of glycosylation pattern and selective pressure

Potential *N*-glycosylated sites are illustrated by light dashed rectangles in Figs. 3 and 4. The NA1 Cameroonian strains showed four predicted sites of *N*-glycosylation. The first site is located at position 237 with respect to reference strain A2. One Cameroonian sequence has lost this glycosylation site due to the substitution N237D. The third *N*-glycosylation site is located at position 273. The two remaining *N*-glycosylation sites shared by all Cameroonian sequences were located at positions 251 and 294. Two putative *N*-glycosylation sites (AA 296 and 310) have been identified within HRSV-B Cameroonian strains. The BA-9b genotype showed one additional *N*-glycosylation site located at residue 230. This additional *N*-glycosylation was due to the specific K233T substitution for this genotype. The number of serine and threonine residues, probably *O*-glycosylated, was 48 for HRSV-B and ranged from 2 to 4 for HRSV-A. In the HVR2 region of the *G* gene for HRSV-B Cameroonian strains, five sites under positive selection were found at positions I200, L219, T270, V271, and H287. Four sites under negative selection were located at P216, L252, P295, and T302. HRSV-A sequences of this study have not been analyzed for sites under selective pressure due to their low number.

## Discussion

We performed molecular characterization of the HVR2 region of the *G* gene for HRSV sequences collected from Cameroonian patients in Yaounde, Cameroon. This study was carried out during three epidemic seasons, from September 2011 to December 2013. As previously described in Cameroon, the HRSV detection was observed during the major rainy season from September to December [33]. This seasonality of HRSV is also consistent with that reported in Gabon and the Central African Republic, two Cameroonian neighboring countries [41, 42]. Because of a low viral load, we were not able to amplify some HRSV samples (19.2%; 11/57) in this study. This result is similar to those reports in other studies [7, 8, 11, 43]. Unlike the global trend recorded from 2011 to 2013, HRSV-B group predominated on HRSV-A in this study [44]. All HRSV-B viruses of this study were clustered with BA-9 with specific duplication of 60 nucleotides in the HVR2 domain. As observed in several other studies, these BA-9 were classified into two subgroups referred as BA-9a and BA-9b [12, 13, 25, 43]. A similar duplication of 72 nucleotides to that of BA genotype was also reported by Eshaghi et al., in Canada in 2010 for the ON-1 genotype, a member of the HRSV-A group [21]. The ON1 genotypes with GA2 and GA5 were predominant genotypes worldwide from 2011 to 2013 while we found only the NA1 genotype in this study [32, 44–46]. The NA1 and BA-9 genotype were also reported in South Africa and Senegal [32, 47]. The *N*-glycosylation sites N237, N251, N273, and N294 previously reported in the HRSV-A genotype NA1 were also observed in the sequences of the current study [5, 13, 19, 21–23, 35]. As previously reported, *N*-glycosylation sites specific for HRSV-B N296 and N310 were observed in all sequences of this study [5, 13, 15, 19, 22, 23, 35, 40]. An additional *N*-glycosylation site, N230, was observed in the BA-9b sequences reported in this work because of the P231S mutation. This additional site has also been reported for BA-10 genotype sequences [5, 40]. The acquisition of this additional *N*-glycosylation site in the BA-9b genotype described in this study could further confer to them a selective advantage. Indeed, it has been shown that variations in the carbohydrate side chain of the G protein may contribute to a change in antigenicity of genotypes through activation or inhibition of binding of specific antibodies [48]. As previously reported, analysis of site-by-site selective pressure in the HVR2 region of the G protein in HRSV-B Cameroonian strains showed five positively selected sites (I200, L219, T270, V271, and H287) and four sites negatively selected (P216, L252, P295, and T302) [10, 26, 47]. This selective pressure can be explained by the antigenicity of the hypervariable C-terminal region of the *G* gene that contains multiple epitopes recognized by neutralizing antibodies [49, 50].

## Conclusion

The current study provides for the first time data on HRSV genotypes circulating in Yaounde, Cameroon. Genetic characterization of HVR2 of HRSV G glycoprotein, a target for many vaccines under development, indicated that two HRSV subtypes (NA-1 and BA-9) co-circulated during this study. This study also points out the importance of carrying out other studies in limited-resource countries such as Cameroon where data remain scarce.

## Abbreviations

AA: Amino acids
HRSV: Human respiratory syncytial virus
HVR: Hypervariable region

## References

[1] Nair H, Nokes DJ, Gessner BD, Dherani M, Madhi SA, Singleton RJ (2010). Global burden of acute lower respiratory infections due to respiratory syncytial virus in young children: a systematic review and meta-analysis. Lancet.

[2] Afonso CL, Amarasinghe GK, Bányai K, Bào Y, Basler CF, Bavari S (2016). Taxonomy of the order Mononegavirales: update 2016. Arch Virol.

[3] Johnson PR, Spriggs MK, Olmsted RA, Collins PL (1987). The G glycoprotein of human respiratory syncytial viruses of subgroups A and B: extensive sequence divergence between antigenically related proteins. Proc Natl Acad Sci U S A.

[4] Sullender WM, Mufson MA, Anderson LJ, Wertz GW (1991). Genetic diversity of the attachment protein of subgroup B respiratory syncytial viruses. J Virol.

[5] Arnott A, Vong S, Mardy S, Chu S, Naughtin M, Sovann L (2011). A study of the genetic variability of human respiratory syncytial virus (HRSV) in Cambodia reveals the existence of a new HRSV group B genotype. J Clin Microbiol.

[6] Dapat IC, Shobugawa Y, Sano Y, Saito R, Sasaki A, Suzuki Y (2010). New genotypes within respiratory syncytial virus group B genotype BA in Niigata, Japan. J Clin Microbiol.

[7] de-Paris F, Beck C, de Souza Nunes L, Machado ABMP, Paiva RM, da Silva Menezes D (2014). Evaluation of respiratory syncytial virus group A and B genotypes among nosocomial and community-acquired pediatric infections in southern Brazil. Virol J.

[8] Hirsh S, Hindiyeh M, Kolet L, Regev L, Sherbany H, Yaary K, et al. Epidemiological changes of respiratory syncytial virus (RSV) infections in Israel. PLoS One. 2014; 10.1371/journal.pone.0090515. 10.1371/journal.pone.0090515 PMC3940902 24594694

[9] Khor C-S, Sam I-C, Hooi P-S, Chan Y-F (2013). Displacement of predominant respiratory syncytial virus genotypes in Malaysia between 1989 and 2011. Infect Genet Evol.

[10] Kushibuchi I, Kobayashi M, Kusaka T, Tsukagoshi H, Ryo A, Yoshida A (2013). Molecular evolution of attachment glycoprotein (G) gene in human respiratory syncytial virus detected in Japan 2008–2011. Infect Genet Evol.

[11] Liu J, Mu Y, Dong W, Yao F, Wang L, Yan H (2014). Genetic variation of human respiratory syncytial virus among children with fever and respiratory symptoms in Shanghai, China, from 2009 to 2012. Infect Genet Evol.

[12] Ohno A, Suzuki A, Lupisan S, Galang H, Sombrero L, Aniceto R (2013). Genetic characterization of human respiratory syncytial virus detected in hospitalized children in the Philippines from 2008 to 2012. J Clin Virol.

[13] Tabatabai J, Prifert C, Pfeil J, Grulich-Henn J, Schnitzler P. Novel respiratory syncytial virus (RSV) genotype ON1 predominates in Germany during winter season 2012–13. PLoS One. 2014; 10.1371/journal.pone.0109191. 10.1371/journal.pone.0109191 PMC4188618 25290155

[14] Tran DN, Pham TMH, Ha MT, Tran TTL, Dang TKH, Yoshida L-M, et al. Molecular epidemiology and disease severity of human respiratory syncytial virus in Vietnam. PLoS One. 2013; 10.1371/journal.pone.0045436. 10.1371/journal.pone.0045436 PMC3551923 23349659

[15] Aamir UB, Alam MM, Sadia H, Zaidi SSZ, Kazi BM. Molecular characterization of circulating respiratory syncytial virus (RSV) genotypes in Gilgit Baltistan Province of Pakistan during 2011–2012 winter season. PLoS One. 2013; 10.1371/journal.pone.0074018. 10.1371/journal.pone.0074018 PMC3772930 24058513

[16] Mufson MA, Orvell C, Rafnar B, Norrby E (1985). Two distinct subtypes of human respiratory syncytial virus. J Gen Virol.

[17] Peret TC, Hall CB, Schnabel KC, Golub JA, Anderson LJ (1998). Circulation patterns of genetically distinct group A and B strains of human respiratory syncytial virus in a community. J. Gen. Virol..

[18] Peret TCT, Hall CB, Hammond GW, Piedra PA, Storch GA, Sullender WM (2000). Circulation patterns of group A and B human respiratory syncytial virus genotypes in 5 communities in North America. J Infect Dis.

[19] Baek YH, Choi EH, Song M-S, Pascua PNQ, Kwon H, Park S-J (2012). Prevalence and genetic characterization of respiratory syncytial virus (RSV) in hospitalized children in Korea. Arch Virol.

[20] Cui G, Zhu R, Qian Y, Deng J, Zhao L, Sun Y, et al. Genetic variation in attachment glycoprotein genes of human respiratory syncytial virus subgroups A and B in children in recent five consecutive years. PLoS One. 2013; 10.1371/journal.pone.0075020. 10.1371/journal.pone.0075020 PMC3775769 24069376

[21] Eshaghi A, Duvvuri VR, Lai R, Nadarajah JT, Li A, Patel SN, et al. Genetic variability of human respiratory syncytial virus a strains circulating in Ontario: a novel genotype with a 72 nucleotide G gene duplication. PLoS One. 2012; 10.1371/journal.pone.0032807. 10.1371/journal.pone.0032807 PMC3314658 22470426

[22] Shobugawa Y, Saito R, Sano Y, Zaraket H, Suzuki Y, Kumaki A (2009). Emerging genotypes of human respiratory syncytial virus subgroup A among patients in Japan. J Clin Microbiol.

[23] Auksornkitti V, Kamprasert N, Thongkomplew S, Suwannakarn K, Theamboonlers A, Samransamruajkij R (2013). Molecular characterization of human respiratory syncytial virus, 2010–2011: identification of genotype ON1 and a new subgroup B genotype in Thailand. Arch Virol.

[24] Blanc A, Delfraro A, Frabasile S, Arbiza J (2004). Genotypes of respiratory syncytial virus group B identified in Uruguay. Arch Virol.

[25] Gimferrer L, Andrés C, Campins M, Codina MG, Rodrigo JA, Melendo S, et al. Circulation of a novel human respiratory syncytial virus group B genotype during the 2014–2015 season in Catalonia (Spain). Clin. Microbiol. Infect. Off. Publ. Eur. Soc. Clin. Microbiol Infect Dis. 2015; 10.1016/j.cmi.2015.09.013. 10.1016/j.cmi.2015.09.013 26408279

[26] Ren L, Xiao Q, Zhou L, Xia Q, Liu E (2015). Molecular characterization of human respiratory syncytial virus subtype B: a novel genotype of subtype B circulating in China. J Med Virol.

[27] Trento A, Galiano M, Videla C, Carballal G, García-Barreno B, Melero JA (2003). Major changes in the G protein of human respiratory syncytial virus isolates introduced by a duplication of 60 nucleotides. J. Gen. Virol..

[28] Trento A, Viegas M, Galiano M, Videla C, Carballal G, Mistchenko AS (2006). Natural history of human respiratory syncytial virus inferred from phylogenetic analysis of the attachment (G) glycoprotein with a 60-nucleotide duplication. J Virol.

[29] Ábrego LE, Delfraro A, Franco D, Castillo J, Castillo M, Moreno B, et al. Genetic variability of human respiratory syncytial virus group B in Panama reveals a novel genotype BA14. J Med Virol. 2017; 10.1002/jmv.24838. 10.1002/jmv.24838 28464479

[30] PATH (2017). RSV vaccine and mAb snapshot—PATH vaccine resource library.

[31] Agoti CN, Otieno JR, Ngama M, Mwihuri AG, Medley GF, Cane PA (2015). Successive respiratory syncytial virus epidemics in local populations arise from multiple variant introductions, providing insights into virus persistence. J Virol.

[32] Fall A, Dia N, Cisse EHAK, Kiori DE, Sarr FD, Sy S (2016). Epidemiology and molecular characterization of human respiratory syncytial virus in Senegal after four consecutive years of surveillance, 2012–2015. PLoS One.

[33] Njouom R, Yekwa EL, Cappy P, Vabret A, Boisier P, Rousset D (2012). Viral etiology of influenza-like illnesses in Cameroon, January–December 2009. J Infect Dis.

[34] Kenmoe S, Tchendjou P, Vernet M-A, Moyo-Tetang S, Mossus T, Njankouo-Ripa M (2016). Viral etiology of severe acute respiratory infections in hospitalized children in Cameroon, 2011–2013. Influenza Other Respir Viruses.

[35] Forcic D, Ivancic-Jelecki J, Mlinaric-Galinovic G, Vojnovic G, Babic-Erceg A, Tabain I (2012). A study of the genetic variability of human respiratory syncytial virus in Croatia, 2006–2008. J Med Virol.

[36] Tamura K, Stecher G, Peterson D, Filipski A, Kumar S (2013). MEGA6: molecular evolutionary genetics analysis version 6.0. Mol Biol Evol.

[37] Thompson JD, Higgins DG, Gibson TJ (1994). CLUSTAL W: improving the sensitivity of progressive multiple sequence alignment through sequence weighting, position-specific gap penalties and weight matrix choice. Nucleic Acids Res.

[38] Julenius K, Mølgaard A, Gupta R, Brunak S (2005). Prediction, conservation analysis, and structural characterization of mammalian mucin-type O-glycosylation sites. Glycobiology.

[39] Pond SLK, Frost SDW (2005). Not so different after all: a comparison of methods for detecting amino acid sites under selection. Mol Biol Evol.

[40] Tuan TA, Thanh TT, Hai NT, LBB T, LTN K, LAH D (2015). Characterization of hospital and community-acquired respiratory syncytial virus in children with severe lower respiratory tract infections in Ho Chi Minh City, Vietnam, 2010. Influenza Other Respir Viruses.

[41] Lekana-Douki SE, Nkoghe D, Drosten C, Ngoungou EB, Drexler JF, Leroy EM (2014). Viral etiology and seasonality of influenza-like illness in Gabon, March 2010 to June 2011. BMC Infect Dis.

[42] Nakouné E, Tricou V, Manirakiza A, Komoyo F, Selekon B, Gody JC (2013). First introduction of pandemic influenza A/H1N1 and detection of respiratory viruses in pediatric patients in Central African Republic. Virol J.

[43] Gimferrer L, Campins M, Codina MG, Martín Mdel C, Fuentes F, Esperalba J (2015). Molecular epidemiology and molecular characterization of respiratory syncytial viruses at a tertiary care university hospital in Catalonia (Spain) during the 2013–2014 season. J Clin Virol.

[44] Hause AM, Henke DM, Avadhanula V, Shaw CA, Tapia LI, Piedra PA (2017). Sequence variability of the respiratory syncytial virus (RSV) fusion gene among contemporary and historical genotypes of RSV/A and RSV/B. PLoS One.

[45] Otieno JR, Kamau EM, Agoti CN, Lewa C, Otieno G, Bett A (2017). Spread and evolution of respiratory syncytial virus a genotype ON1, coastal Kenya, 2010–2015. Emerg Infect Dis.

[46] Valley-Omar Z, Muloiwa R, Hu N-C, Eley B, Hsiao N-Y (2013). Novel respiratory syncytial virus subtype ON1 among children, Cape Town, South Africa, 2012. Emerg Infect Dis.

[47] Pretorius MA, van Niekerk S, Tempia S, Moyes J, Cohen C, Madhi SA (2013). Replacement and positive evolution of subtype A and B respiratory syncytial virus G-protein genotypes from 1997–2012 in South Africa. J Infect Dis.

[48] Palomo C, Cane PA, Melero JA (2000). Evaluation of the antibody specificities of human convalescent-phase sera against the attachment (G) protein of human respiratory syncytial virus: influence of strain variation and carbohydrate side chains. J Med Virol.

[49] Cane PA (1997). Analysis of linear epitopes recognised by the primary human antibody response to a variable region of the attachment (G) protein of respiratory syncytial virus. J Med Virol.

[50] Norrby E, Mufson MA, Alexander H, Houghten RA, Lerner RA (1987). Site-directed serology with synthetic peptides representing the large glycoprotein G of respiratory syncytial virus. Proc Natl Acad Sci U S A.

